# Everybody's Page

**Published:** 1889-08-31

**Authors:** 


### Everybody's Page.

Formerly typhus fever used to visit this country as an annual epidemic, and killed one out of every three it attacked.

The first elephant ever seen in England was sent as a present to Henry III. by the King of France about the year 1255. Numbers of people crowded to see the curiosity, and a special building was erected for it at the Tower.

The newsboys of Melbourne should be superior to those of London. The Newsboys' Brigade there has a club and library of its own. Evening classes are given there, and the members of the brigade were invited by Mr. Santley to one of his recent concerts.

The size of the pupil of the eye varies greatly in different persons. In those of a sanguine temperament the diameter may not exceed an eighth of an inch, while in those of a sluggish disposition it is twice as large. This measurement is calculated on the average size of the pupil, independent of the involuntary contractions caused by keener light.

It is supposed that certain diseases are so antagonistic to each other that a patient who suffers from one cannot take the other. Such are tuberculosis and rheumatism, and the experiment has been made of turning a consumptive patient into a rheumatic by means of inoculation with the blood of a rheumatic fever sufferer, by way of curing the original disease.

Salt Lake City has recently won high praise as a sanatorium. It combines the advantages of a seaside with those of a mountain resort, and has sulphur springs thrown in. It has the disadvantage, however—not counting the risk of imbibing peculiar social ideas along with the ozone—of having no proper system of drainage. But the wily Mormons are making arrangements to improve their city in that respect.

There is something characteristically feminine in the fact that the author of the "Recollections of a Nurse," while she speaks calmly of the painful sights she witnessed in the course of her work, should record with evident distaste an interview with certain cockroaches. Although she did not faint as her assistant did, it evidently was rather a strain on her moral courage to avoid doing so. There is something in the constitution of women, or cockroaches, which causes an ineradicable antipathy between them.

Dr. Arnaudet, who practises medicine in Normandy, has come to the conclusion that cancer is contagious, and that the germ of the disease, a special microbe, may be conveyed by water. In several cases that came under his notice the patients were in the habit of drinking cider which had been made with impure water. It seems that it is a custom to use water taken from swampy ground in the manufacture of cider, and the darker in colour the water is, therefore the more impure, the more highly is it valued for adding to the apple juice.

Commercial or artificial vanillin, which is made, not from the vanilla bean but from the cambium sap of the pine, threatened at one time to injure the value of the genuine product, for one ounce of vanillin is equal in strength to forty ounces of good vanilla beans. But it has been found that the flavour of the artificial product is very evanescent, so, though it can be used in domestic cookery, where the dainty it is used in the concoction of is to be consumed within a day, it is useless for chocolate or other confectionery in which a certain permanence of odour and taste is necessary. In this, as in most things, Nature holds her own.

One essential in speaking in a large room or hall is to pitch the voice high, and to take care that it does not drop at the end of a word or sentence.

The missionaries have succeeded in getting rid of "Ava," the national intoxicant of the South Sea Islands. Perhaps they were aided by the fact that, according to Captain Wilkes, the taste resembled that of rhubarb and magnesia

Dr. May, of San Francisco, when called on to give witness as a medical expert in a murder case declined to speak unless he was paid in advance. For which piece of shrewdness he was sentenced to six months' imprisonment for contempt of court.

At a time when Abraham Lincoln, as President of the United States, was being pestered with requests for offices and anything else he could bestow, he fell ill, and was informed that he was suffering from varioloid. "Is it infectious?" he asked, and being told it was, he remarked "At last I've got something I can give everybody."

A large number of defects of hearing were set down in olden times to "worms in the ear." The poor harmless earwig has been labelled according to its supposed crimes, though its Scottish name of "clipshear" is much more descriptive of its appearance and habits; and some old-fashioned folks cannot yet be brought to believe that blackbeetles do not lodge in their ears and cause deafness.

A case is recently reported in which a boy, suffering from mumps, infected his pet dog. The psychological effects are the most curious symptoms of the case. Originally the boy was wild and pugnacious, while the dog was gentle and fond of home. Now the boy has become quiet and unassuming, willing to "live and let live," while the dog is boisterous, and goes about a great deal, challenging all the canines of the neighbourhood to mortal combat. How have they changed natures so completely?

It is a pity that something has not been discovered to account for the bad name belonging to the east wind. An east wind is usually a cold dry wind, but why should it have such an evil effect on people who cannot merely endure but enjoy a dry cold north-wester? There must be some specific quality about the east wind that makes it so depressing. No actual physical symptoms are produced in those most sensitive to this wind by its presence, yet it causes a thorough prostration of mind and body, and a warm east wind, which we sometimes get in summer, is, if possible, even more depressing than a cold one.

Dr. Eccles, of Brooklyn, has lately taken on him to prove that the man who walks uprightly brings endless troubles on himself. It may be well to state that it is physical, not moral, uprightness that is so mischievous, and that the resulting woes are bodily ones. Our remote ancestors, says Dr. Eccles, were mammalian quadrupeds, and their anatomy was adapted to this mode of progression. When man, proud man, began to look up and walk on his hind legs, he disarranged the anatomical balance that had hitherto kept him in health, and pathological changes result. Thence come varicose veins and ulcers, bow legs, goitre, tumours, and various other diseases, all due, it appears to the upright posture. Yet we think that it would be easy to find other and nearer causes for all these ills, which it is not by any means certain would be cured by returning to the quadrupedal mode of progression. In fact, the quadrupeds we know are by no means free from them. Take the bow legs, for example. Has Dr. Eccles ever seen a dachshund?

